# Quality Maintenance and Increased Firmness of Fresh‐Cut Strawberries Using Vacuum Infusion of Calcium Lactate and Pectin Methylesterase

**DOI:** 10.1111/1750-3841.70658

**Published:** 2025-11-17

**Authors:** Alysson Caetano Soares, Mônica Silva de Jesus, Steven A. Sargent, Jeffrey K. Brecht, Luiz Fernando Ganassali de Oliveira Junior, Marcelo Augusto Gutierrez Carnelossi

**Affiliations:** ^1^ Fruit and Vegetables Laboratory, PPGAGRI, Department of Food Engineering Federal University of Sergipe (UFS) São Cristóvão Sergipe Brazil; ^2^ Laboratory of Flavor and Chromatographic Analysis, Federal University of Sergipe São Cristóvão Sergipe Brazil; ^3^ Horticultural Sciences Department University of Florida/IFAS Gainesville Florida USA

**Keywords:** enzyme activity, firming, *Fragaria* × *ananassa*, postharvest

## Abstract

The demand for healthy foods has led to an increase in the consumption of fresh fruits, especially fresh‐cut (FC) fruits. However, FC processing negatively affects fruit firmness, an important parameter for consumer acceptance. The objective of this work was to evaluate the effectiveness of the combined use of vacuum, calcium lactate, and pectin methylesterase (PME) to increase the shelf life and quality of FC strawberries. FC strawberry halves were subjected to vacuum infusion with water, PME (10UI), PME plus 1% calcium lactate, or 1% calcium lactate alone; FC strawberries without infusion were used as the control. Firmness, loss of fresh mass, anthocyanin, color, soluble solids content (SSC), titratable acidity (TA) SSC/TA ratio, pH, visible spoilage and damage, visual quality, galacturonic acid content, degree of methylation, electrolyte efflux, PME activity, and respiration were measured initially and every 3 days for 9 days during 5°C storage. Vacuum infiltration of PME plus calcium lactate increased firmness by 90.7% for LCa + PME and 119.8% for LCa on Day 3, delayed weight loss to below 0.8% for all except PME treatments, maintained freshness and anthocyanin content, reduced electrolyte efflux by 38% compared to the control, and lowered respiration rates to 134.92 and 135.62 mg kg^−1^ h^−1^ CO_2_ for LCa + PME and LCa, respectively, whereas the control reached 163.36 mg kg^−1^ h^−1^ CO_2_. Additionally, it minimized fruit damage. Therefore, vacuum infusion of 1% Lca + PME provided promising applications in extending the shelf life of cut strawberries by up to 3 days.

**Practical Applications:**

This research offers a practical solution for the fresh‐cut fruit industry by improving the firmness and shelf life of strawberries through vacuum infusion with calcium lactate and PME. By maintaining texture, reducing weight loss, and slowing respiration, this method can help retailers provide fresher, higher‐quality strawberries with an extended shelf life of up to 3 days. Consumers would benefit from better‐tasting, longer‐lasting fresh‐cut fruit, reducing waste, and enhancing convenience.

## 1 Introduction

The growing demand for healthy, safe, and convenient foods has driven an increased consumption of fresh fruits and vegetables, which provide essential nutrients, support balanced diets, and are associated with reduced risks of chronic diseases like Type 2 diabetes and certain cancers (Aune et al. 2017). Furthermore, the relationship between a healthy diet, adequate nutritional status, and strengthening the immune system has been highlighted as a relevant factor for the consumer (Gonella et al. 2022), with strawberries considered an important component in this context.

Maintaining the nutritional quality and safety of fresh products is a challenge for the food industry. Given the demand for fresh products, the application of minimal or fresh‐cut (FC) processing has been investigated for many fruits and vegetables, such as apple (Perinban et al. 2022), dragon fruit (X. Li et al. 2022), kiwi (Hu et al. 2022), carrot (L. Li et al. 2021), yam (Teoh et al. 2016), mango (Kirtil et al. 2014), and strawberry (Méndez‐Galarraga et al. 2022). Strawberry (*Fragaria × ananassa*), belonging to the Rosacea family, is widely consumed fresh due to its aroma, color, and pleasant flavor. However, FC processing can negatively alter the firmness of the fruits by accelerating cell wall degradation and loss of turgidity (Pinheiro and Almeida 2008).

The decrease in firmness following FC fruit processing is associated with modifications and degradation of cell wall components such as cellulose, hemicellulose, and most notably pectins, due to their abundance and high sensitivity to chemical reactions. The enzymes pectin methylesterase (PME) and polygalacturonase (PG) play key roles in pectin breakdown. PME catalyzes the removal of methyl ester groups, exposing negatively charged carboxyl groups that serve as substrates for PG‐mediated depolymerization (Li et al. 2024). Demethylation of pectin by PME exposes negatively charged carboxylic acid groups that can subsequently bind calcium ions. These de‐esterified pectins can bind calcium ions, forming cross‐links that strengthen the pectin network and reinforce cell wall structure, contributing to improved texture retention during storage (Li et al. 2024; Kirtil et al. 2014). As a result, calcium treatments can reduce softening, extend shelf life (Sirijariyawat et al. 2012), and consequently improve consumer acceptance of FC fruit.

Although calcium chloride (CaCl_2_) is commonly applied postharvest (Langer et al. 2019; Prajapati et al. 2021), its sensory drawbacks, especially bitterness to residual chlorine (Luna‐Guzmán and Barrett. 2000), can limit consumer acceptance and commercial viability. Calcium lactate, a compound with Generally Recognized As Safe (GRAS) status (US FDA, 21 CFR § 184.1207), has been proposed as a viable alternative, offering comparable structural benefits with fewer sensory issues (Hu et al. 2022). Luna‐Guzmán and Barrett (2000) demonstrated that calcium lactate maintained superior firmness in FC produce compared to calcium chloride, without inducing the undesirable bitterness associated with chloride‐based treatments. The firming effect of calcium can be further enhanced by exogenous PME, which increases calcium binding sites via pectin demethylation (Huang et al. 2023). The effect of applying calcium and/or exogenous PME on firmness has been evaluated in different fruits such as papaya (Yang et al. 2017), mango (Kirtil et al. 2014), strawberry (Carnelossi et al. 2018), apricot (Liu et al. 2017), guava (Werner et al. 2009), and others.

Vacuum infusion has been used to improve the quality, physical–chemical characteristics, and mainly the firmness of FC products, which, in porous matrices, allows the exchange of gases and liquids with an impregnation solution under the action of the hydrodynamic mechanism, enabling the transfer of compounds of interest, such as PME, to intercellular spaces (Derossi et al. 2013; Guillemin et al. 2008). Combined infusion of PME and calcium has been reported to improve the firmness of fresh mangoes (Sirijariyawat et al. 2012). In the same way, the infusion of CaCl_2_ and PME into raspberries has been verified to maintain the fruit firmness (Yan et al. 2022). In addition, the isolated application of PME by vacuum impregnation was observed to result in an increase in the rigidity of pectins and the mechanical rigidity of apple tissue by Guillemin et al. (2008). In view of the above, the objective of this study was to evaluate the effectiveness of the combined use of vacuum, calcium lactate, and PME to increase the shelf life and quality of FC strawberries.

## 2 Materials and Methods

### 2.1 Plant Material

The strawberries (*Fragaria × ananassa*) were purchased from a local store at the full commercial development stage, with more than 75% red color (PBMH and PIMo 2009) and were transported to the Fruit and Vegetable Laboratory of the Department of Food Technology at the Federal University of Sergipe.

### 2.2 FC Processing

Whole strawberries were selected based on physical integrity, appearance and absence of mechanical damage (Carnelossi et al. 2018). The fruit were previously washed with running water to remove residues. Both the removal of the calyx (3 mm from its base) and a longitudinal cut were performed manually with the aid of a sharp blade. Sanitization was carried out by immersion of strawberry halves in chlorinated water cooled to 5°C ± 0.5°C at a concentration of 200 ppm for 10 min (Carnelossi et al. 2018). After the initial chlorine submersion, additional rinsing was carried out in 5 ppm chlorinated water cooled to 5°C  ± 0.5°C with ice for 3 min. After rinsing, the strawberry halves were placed in sanitized sieves to drain in a controlled environment at the processing temperature 5°C ± 0.5°C under 79% ± 2% RH (Carnelossi et al. 2018).

### 2.3 Vacuum Infusion of FC Strawberry

Vacuum infusion was performed in a desiccator equipped with a pressure gauge and connected to a vacuum pump, where pressure was rapidly reduced to 12 kPa. Strawberry halves (100 g) in 250 mL beakers with 125 mL of solution (5°C ± 0.5°C) underwent 10 min of vacuum exposure, followed by 1 min of gradual atmospheric pressure restoration. The following treatments were carried out: control (without infusion); infusion with water only; infusion with aqueous solution containing commercial PME; infusion with an aqueous solution containing commercial PME and calcium lactate (1%); and an aqueous solution containing only calcium lactate. The commercial PME enzyme (NovoShape) used has a declared activity of 10 PEU mL^−1^, manufactured by Novozymes, Denmark. The concentrations of PME and calcium lactate (C_6_H_10_CaO_6_) were each 1% as used previously by Carnelossi et al. (2018).

The FC strawberries were divided into five treatments, namely, FC fruit without infusion (C), FC fruit infused with water (W), FC fruit infused with commercial PME, FC fruit infused with PME and calcium lactate (PME +  LCa—1%), and FC fruit infused with only calcium lactate (LCa).

After the infusion process, the solution was drained using domestic sieves before being packaged and stored. After infusion, pieces were packaged in polypropylene packaging and stored in a vertical display with air circulation (Springer) at a temperature of 5°C ± 1°C for 6 days, under a relative humidity of 79%. Samples were collected every 3 days to carry out biochemical and physical–chemical analyses, being previously crushed and homogenized with the aid of the Sorvall Omnimixer equipment and stored at −18°C until the moment of analysis. Analyses to determine pH, titratable acidity (TA), soluble solids content (SSC), firmness, color, visible spoilage, freshness, damage, and electrolyte efflux, as well as physiological analyses, were carried out on the fresh sample, that is, before the freezing process.

### 2.4 Firmness

The firmness of the FC fruit halves was measured using a digital penetrometer (TR Turoni, model 53205, Forli, Italy), with an 8‐mm diameter tip according to the manufacturer. The results were expressed in Newtons (N).

### 2.5 Weight Loss

Weight loss was determined during storage by subtracting the sample weight from their previously recorded initial weight of storage (Day 0) and expressed as % of weight loss compared to the initial fruit weight (Zainal et al. 2019):

$$\mathrm{Weight}\;\mathrm{loss}\;\left(\%\right)=\frac{\mathrm{Fruit}\;\mathrm{initial}\;\mathrm{weight}-\;\mathrm{fruit}\;\mathrm{weight}}{\mathrm{Fruit}\;\mathrm{initial}\;\mathrm{weight}}\;\times 100$$

### 2.6 Electrolyte Efflux

Cellular integrity analysis was performed according to the methodology described by Villalta and Sargent (2004). The strawberry mesocarp was minimally cut into slices (5 mm × 2 mm), 2 g of this tissue was rinsed with distilled water, dried on paper and placed in closed glass tubes with 35 mL of 0.4 M isotonic mannitol solution, and kept at 23°C for 4 h. The conductivity of the bath solution was measured after 4 h of incubation at 23°C by a conductivity meter (Model 3403, Yellow Springs, OH, USA). The total electrolyte content was determined after freezing for 24 h at −20°C, thawing for 1 h at 23°C, and then heating the pieces in a boiling water bath for 30 min. Electrolyte efflux of the fresh tissue was expressed as a percentage of total tissue electrolytes after freezing, thawing, and boiling.

### 2.7 Respiratory Rate

The physiological analysis was carried out according to the methodology described by Furtado et al. (2018) with adaptations. The closed system CO_2_ analysis was performed using approximately 25 g of FC strawberries. The respiratory rate was determined in glass bottles of approximately 120 mL previously prepared with rubber caps. The bottles were closed hermetically and aliquots of 0.1 cm^3^ were removed after 0.5 h of closing the bottles with the aid of a 1000‐µL disposable syringe to quantify the gas composition. The determination of sampling time was carried out experimentally. The analysis of accumulated CO_2_ was carried out on a gas chromatograph (Varian Model CP‐3380), equipped with a thermal conductivity detector (TCD) and an Rt capillary column‐Q‐BOND (RESTEK) (30 m × 0.32 mm ID × 10 µm). The carrier gas used was Helium, with a flow of 5 mL min^−1^. The temperatures of the column, injector, and TCD detector were 30°C, 200°C, and 120°C, respectively. The chromatographic analysis time was 120 s, and the CO_2_ retention time was 60 s.

Quantification of CO_2_ was done by comparing the peak areas of the samples obtained in the Software (Galaxie Workstation) coupled to the chromatograph, with peak areas of the CO_2_ standard of known concentration. The results were expressed in %CO_2_. The %CO_2_ was converted to respiration rate (mL kg^−1 ^h) by calculation from the tissue weight, closure time, and the void space in the bottle.

### 2.8 Isolation of Alcohol Insoluble Solids (AISs)

Isolation of AISs was isolated according to the method described by Huber (1984). Strawberry tissue (20 g) was homogenized in 80 mL of 100% ethanol for 3 min using a Sorvall Omnimixer. The homogenate was refluxed for 30 min in a boiling water bath. When necessary, 100% ethanol was added to maintain ethanol at the initial level. It was then stored overnight at −20°C. The suspension was filtered through a 0.6 µm retention capacity glass microfiber filter (MN GF‐3, 55.5 mm, Macherey‐Nagel, Germany) in an aspiration bottle, followed by washing with 200 mL of 80% ethanol under slow aspiration. Then, it was washed with 100 mL of 100% acetone. The acetone was removed by vacuuming and the powder was air dried in an oven (39°C) for 1 day.

### 2.9 Degree of Methylation (DM) and Galacturonic Acid

Methanol content was determined following the method of Wood and Siddiqui (1971), with modifications. Briefly, 5 mg of AISs were dissolved in 2 mL of distilled water and sonicated for 10 min. Then, 0.8 mL of NaOH (2 M) was added for saponification, followed by incubation at 20°C for 1 h with occasional shaking. Neutralization was performed with 0.8 mL of HCl (2 M), and the mixture was kept at 25°C for 15 min (Ng and Waldron 1997). For quantification, 1 mL of the sample or methanol standard was transferred to chilled test tubes containing 1 mL of H_2_SO_4_ (0.1 N). Next, 0.2 mL of KMnO_4_ (2%, w/v) was added, and the mixture was gently stirred in an ice bath for 15 min. Then, 0.2 mL of sodium arsenite solution (0.5 M in H_2_SO_4_ 0.12 N) and 0.6 mL of distilled water were added, and the reaction was allowed to proceed at room temperature for 1 h. Finally, 2 mL of a 0.02‐M acetylacetone solution in ammonium acetate (2.0 M) and acetic acid (0.05 M) was added, and the mixture was heated at 58–60°C for 15 min.

Total galacturonic acid content was measured according to Ahmed and Labavitch (1978). AIS (5 mg) was homogenized in 2 mL of cold H_2_SO_4_ in a 20‐mL beaker, followed by gradual addition of 0.5 mL of chilled distilled water under magnetic stirring for 5 min. The volume was then adjusted to 20 mL. For analysis, 0.5 mL of the sample was mixed with 3.6 mL of cold tetraborate in H_2_SO_4_ solution, heated in a boiling water bath for 5 min, and cooled under running water. The DM was calculated as the molar ratio between methanol released and total galacturonic acid and expressed as a percentage.

### 2.10 PME Activity

The FC fruit tissue (1.00 g) was crushed in a pre‐cooled (4°C) mortar and homogenized with 6 mL of 8.8% NaCl containing 10 g L^−1^ of PVPP (polyvinylpolypyrrolidone). After extraction at 4°C for 1 h, the solution was centrifuged at 12,000 × *g* for 20 min (Ren et al. 2020). The supernatant was obtained and used to measure enzyme activity. The PME activity was evaluated as described by Vicente et al. (2005). Activity was measured in a mixture containing 0.6 mL of 0.5% pectin (w/v), 0.15 mL of 0.05% bromothymol blue, 0.1 mL of distilled water, and 0.1 mL of enzyme extract (supernatant). The mixture was incubated at 37°C for 30 min and the absorbance at 620 nm was recorded. Enzymatic activity was defined as a reduction of 0.01 units of OD 620 nm min^−1^ kg^−1^ of tissue.

### 2.11 Visual Quality (Freshness, Visible Spoilage, and Mechanical Damage)

The evaluation of FC fruit halves for visual appearance (freshness), visible spoilage, and mechanical damage was performed using methodologies adapted from Jacomino et al. (2011). For freshness, randomly selected FC strawberry halves were evaluated independently by the authors using the following standardized scale: 9 = Excellent: completely fresh appearance, high gloss; 7 = Good: still fresh appearance, shiny; 5 = Fair: not fresh appearance, low brightness, marketability limit; 3 = Poor: dull appearance, usability limit; 1 = Extremely poor: wrinkled appearance. Results were calculated as mean scores for each experimental replicate. Visible spoilage was assessed by enumerating strawberry halves exhibiting postharvest rot or visible fungal growth. The incidence was expressed as a percentage relative to the total number of FC strawberry halves. Mechanical damage was quantified by counting fruit halves displaying postharvest physical injuries, with results expressed as a percentage in relation to the total number of FC fruit halves.

### 2.12 Color

Color parameters were directly measured using a Minolta CR‐300 colorimeter (Konica Minolta Sensing, Osaka, Japan) in the CIE *L* ^*^ *a* ^*^ *b* ^*^ and hue angle (*h*°) value automatically calculated by the instrument's software: *L* ^*^ (lightness): Ranges from 0 (black) to 100 (white); *a*: Red–green axis (+*a* = red, −*a* ^*^ = green); *b*: Yellow–blue axis (+*b* = yellow, −*b* ^*^ = blue); Hue angle (*h*°) = arctan (*b* ^*^/*a* ^*^): Describes color tone (0° = red, 90° = yellow, 180° = green). Measurements were performed according to manufacturer specifications and standardized protocols (Gaya and Sterman Ferraz 2006; HunterLab 2008; Menezes 2012).

### 2.13 Determination of pH, SSC, Titratable Acidity (TA), and RATIO (SSC/TA)

A 5‐g sample of strawberry tissue was weighed, homogenized and mixed with 50 mL of distilled water. The pH of the resulting liquid was measured using the potentiometric method. After calibrating the pH meter, the electrode was immersed in the homogenized aqueous mixture, and the measurement was carried out (Instituto Adolfo Lutz 2008).

The SSC of the strawberry tissue was determined by applying a few drops of juice onto the prism of a digital refractometer model HI 96801 (Hanna Instruments, Romania) according to the official method (AOAC 1997). The results were expressed in °Brix.

The TA of the strawberry tissue was determined using 10 mL of the aqueous homogenate used to determine the pH, adding 1% phenolphthalein indicator and titrating with standardized NaOH (0.1 N) to pH 8.2. The TA was expressed as % citric acid (Instituto Adolfo Lutz 2008).

The ratio of SSC/TA was calculated using the ratio of the °Brix ratio to the percent acidity expressed as % citric acid (Instituto Adolfo Lutz 2008).

### 2.14 Determination of Total Anthocyanin Content

The determination of total anthocyanins was carried out according to Nunes et al. (2006). Aliquots (2 g) of homogenized strawberry tissue were mixed with 18 mL of 0.5% HCl in methanol (v/v). Anthocyanins were extracted by keeping the samples at 4°C for 1 h in the dark. Samples were then filtered using single‐layer fabric (Kimwipe) to remove flocculant. The absorbance of the solution was measured at 520 nm. The results were expressed as mg/100 g fresh weight of pelargonidin‐3‐glucoside.

### 2.15 Statistical Analysis

The design was completely randomized set up in a factorial scheme (5 × 3) with five treatments [FC fruit without infusion (SI); FC fruit infused with water (W); FC fruit infused with PME; FC fruit infused with PME + calcium lactate (PME + LCa—1%); FC fruit infused with only calcium lactate (LCa)] and three evaluation times (0, 3, and 6 days) with four repetitions for each treatment. The results were subjected to analysis of variance (ANOVA) using the *F* test and comparison of means using the Tukey test (*p* < 0.05), expressed as the mean ± standard deviation, with the aid of the computer program SISVAR version 5.8 (Ferreira 2019).

## 3 Results

**FIGURE 1:**
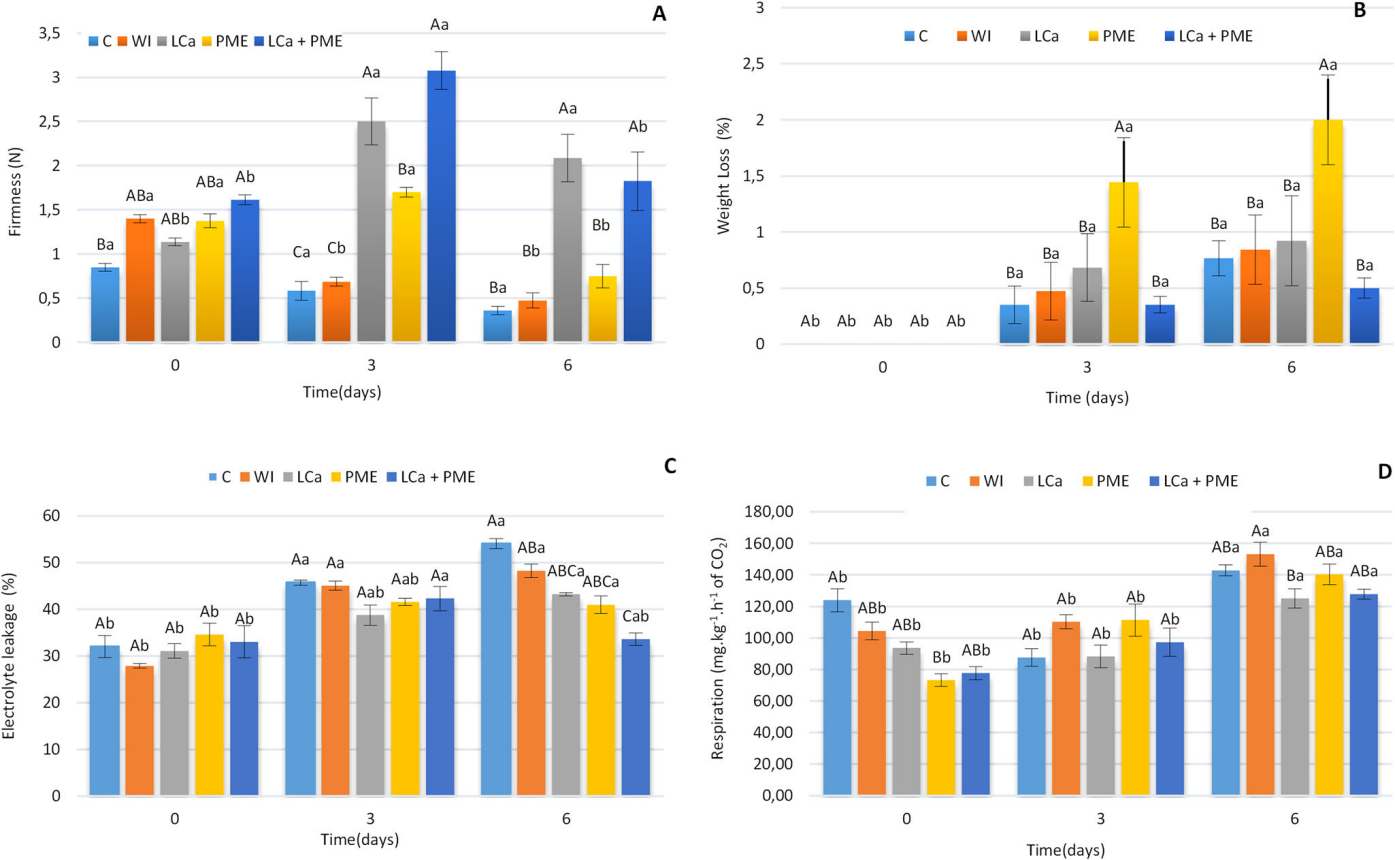
(A) Firmness, (B) weight loss, (C) electrolyte leakage, and (D) respiratory rate of fresh‐cut strawberries stored at 5°C±1°C. C—no treatment and infusion; water—infusion with water; LCa—infusion with calcium lactate; PME—infusion with pectin methylesterase; LCa + PME—infusion with calcium lactate and pectin methylesterase. Different uppercase letters indicate statistical difference between treatments on the same day and different lowercase letters indicate statistical difference between the same treatment across days using the Tukey test (*p* < 0.05). PME, pectin methylesterase.

Fruit halves treated with LCa + PME presented on “Day 0” (Figure 1A) higher firmness values (*p* < 0.05) compared to the control group (C). The treatments with LCa and LCa + PME showed an increase in firmness relative to the pre‐treatment value on the third day of storage (2.5 N for LCa and 3.0 N for LCa + PME), being superior throughout the storage time in relation to the other treatments. A decrease in firmness was observed over time in the control water‐treated samples.

There was an increase in weight loss throughout the storage period for the evaluated treatments, with significant losses observed from the third day of storage (0.66%) (Figure 1B). Greater mass losses were observed in strawberries treated with PME (*p* < 0.05) while for the other treatments, a loss of less than 0.8% was observed over time.

Electrolyte efflux from FC strawberries increased significantly (*p* < 0.05) as a function of time (Figure 1C). However, on the sixth day of storage, it was observed that FC strawberries treated with LCa + PME showed less leakage compared to FC strawberries treated without infusion and with infusion with water alone. Strawberries treated with LCa + PME showed greater electrolyte efflux stability (Figure 1C).

FC strawberries treated with LCa and LCa + PME showed lower respiratory rates (135.62 and 134.92 mg kg^−1^ h^−1^ of CO_2_) than those treated with water alone (163.36 mg kg^−1^ h^−1^ of CO_2_) (Figure 1D). The respiratory rate of FC strawberries did not vary until Day 3, with a significant increase in the respiratory rate only being observed on Day 6 of storage (*p* < 0.05). The effect of treatments did not vary significantly with storage duration (*p* < 0.05).

**FIGURE 2:**
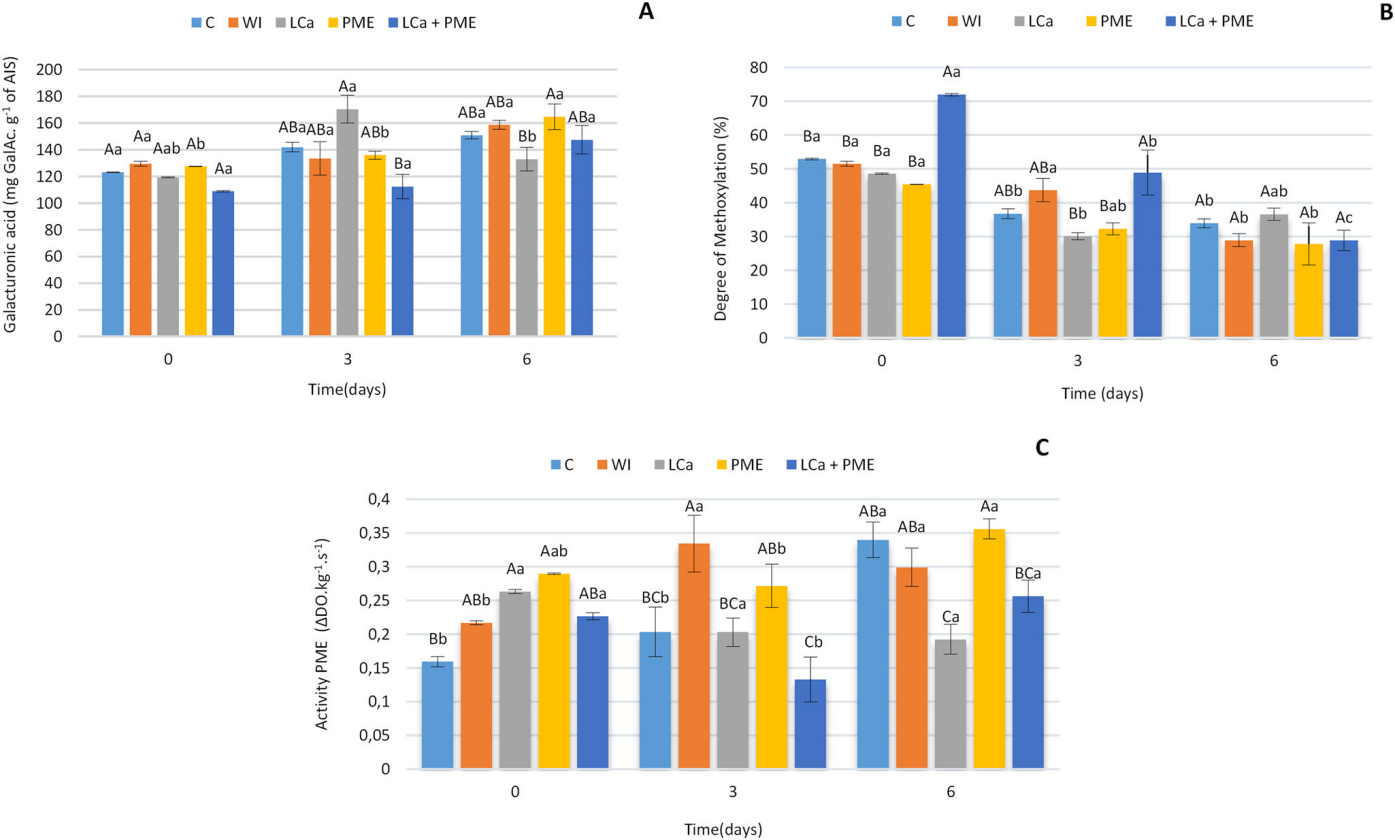
(A) Galacturonic acid, (B) degree of methoxylation, and (C) PME activity of fresh‐cut strawberries stored at 5°C±1°C. C—no treatment and infusion; water—infusion with water; LCa—infusion with calcium lactate; PME—infusion with pectin methylesterase; LCa + PME—infusion with calcium lactate and pectin methylesterase. Different uppercase letters indicate statistical difference between treatments on the same day, and different lowercase letters indicate statistical difference between the same treatment across days using the Tukey test (*p* < 0.05). PME, pectin methylesterase.

There were no significant differences during storage in GalAc acid levels for the C, water, and PME + LCA treatments (*p* < 0.05) (Figure 2A). On Day 3, higher GalAc values were observed for the LCa treatment, differing only from the PME + LCa treatment (Figure 2A).

Higher values of the DM (Figure 2B) for treatments with LCa + PME (72%) were verified on Day 0 in relation to the other treatments (*p* < 0.05) remaining higher on Day 3 of treatments with only LCa and SME. A reduction in the DM was observed in all treatments throughout storage (*p* < 0.05) (Figure 5).

Significant increases (*p* < 0.05) in PME activity were observed during the 6‐day storage period: 113% for control (C), 37% for water‐treated, and 22% for PME‐treated samples (Figure 2C). In contrast, the LCa + PME treatment showed reduced PME activity, reaching its lowest values on Day 3. The LCa treatment maintained consistently lower PME activity throughout storage, showing no significant difference (*p* > 0.05) only when compared to the LCa + PME treatment.

**FIGURE 3:**
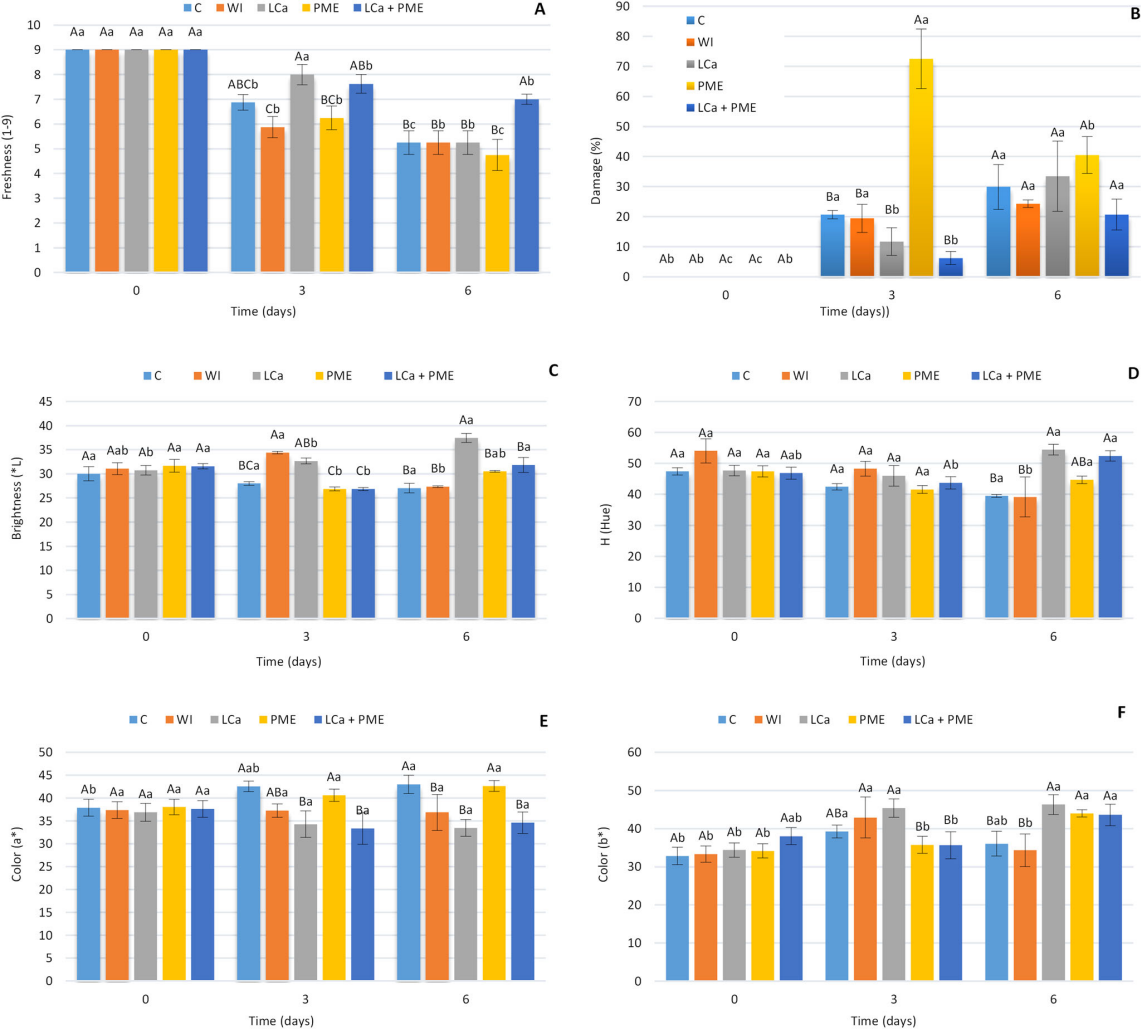
(A) Freshness, (B) damage, (C) brightness (^*^ *L*), (D) hue, (E) color (*a* ^*^), and (F) color (*b* ^*^) of fresh‐cut strawberries stored at 5°C±1°C. C—no treatment and infusion; water—infusion with water; LCa—infusion with calcium lactate; PME—infusion with pectin methylesterase; LCa + PME—infusion with calcium lactate and pectin methylesterase. Different uppercase letters indicate statistical difference between treatments on the same day, and different lowercase letters indicate statistical difference between the same treatment across days using the Tukey test (*p* < 0.05). PME, pectin methylesterase.

**FIGURE 4:**
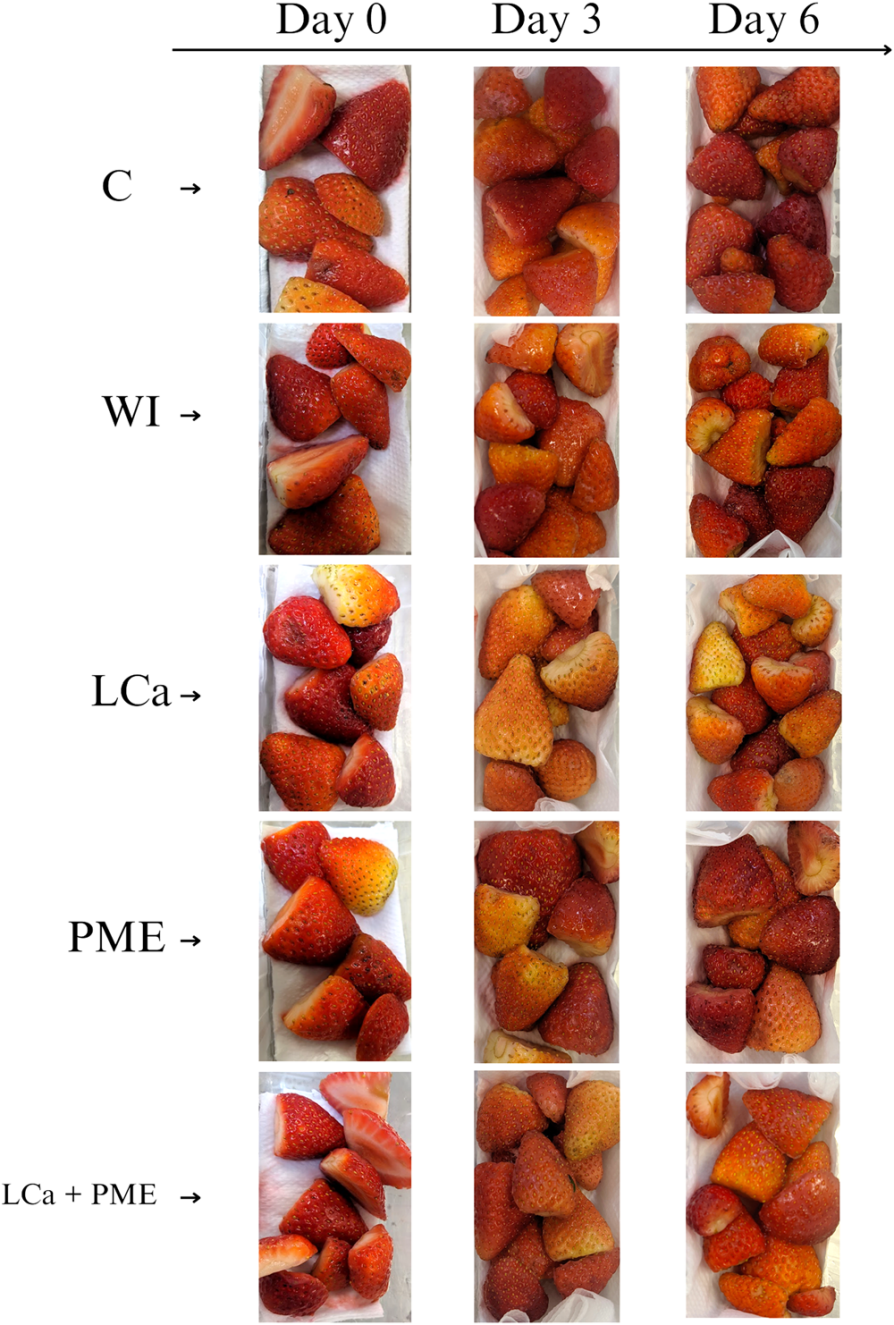
Visual quality of fresh‐cut strawberries stored at 5°C ± 1°C. C—no treatment and infusion; water—infusion with water; LCa—infusion with calcium lactate; PME—infusion with pectin methylesterase; LCa + PME—infusion with calcium lactate and pectin methylesterase. PME, pectin methylesterase.

When evaluating freshness (Figure 3A), a significant reduction was observed in all treatments over time. However, the LCa and LCa + PME samples showed greater freshness compared to samples infused with water alone (Figure 3A). The LCa + PME treatment showed greater freshness retention with a good appearance, fresh and still shiny appearance, maintained throughout the storage time (Figure 4).

**FIGURE 5:**
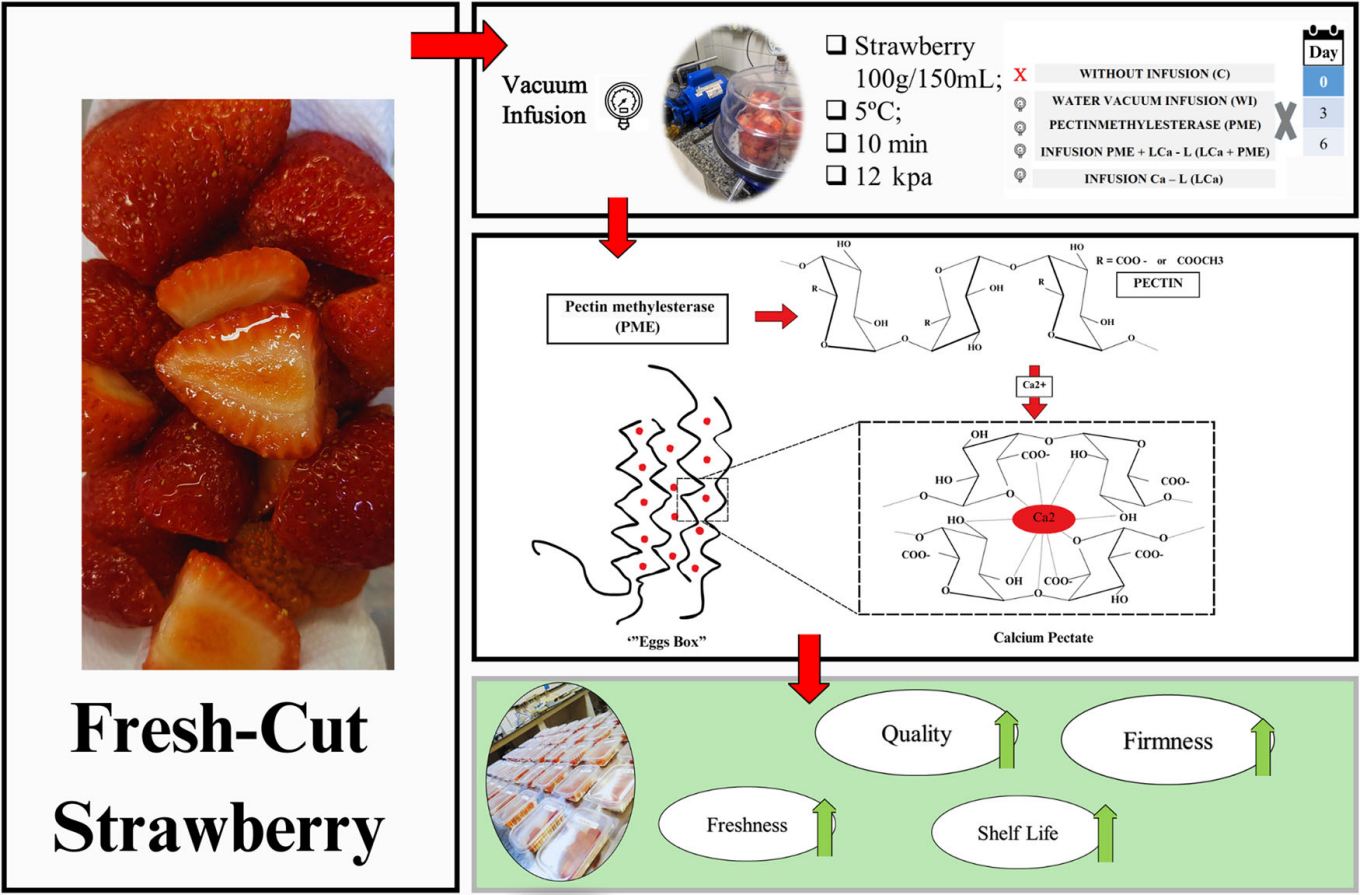
Shelf life extension scheme for fresh cut strawberries with vacuum application of calcium lactate and PME. PME, pectin methylesterase.

There was no significant difference between treatments when evaluating the percentage of visible spoilage of MP strawberries (*p* < 0.05). During storage, an increase in the percentage of damage was also observed. However, for samples treated with calcium sources (LCa and PME + LCa), this variation was smaller (Figure 3B). On the other hand, the C, water and mainly PME treatments showed a high percentage of damage from Day 3 onwards.

When evaluating the color index, there was a reduction in the luminosity (i.e., darkening) of the FC strawberries in the PME and LCa + PME treatments on Day 3 (Figure 3C) (*p* < 0.05), whereas on Day 6, an increase in the *L* ^*^ color index was observed. The *L* ^*^ for FC fruit treated with LCa, was higher than for the other treatments on Day 6 (*p* < 0.05). Furthermore, over time, a significant reduction (reddening) in the Hue* angle (Figure 3D) was observed in fruit treated with water (*p* < 0.05), with higher (less red) values for treatments with LCa and LCa + PME of 54.4. When evaluating the color index *a* ^*^ (Figure 3E), lower levels of the red component were found in FC strawberries treated with calcium, regardless of the day, with values of 34.97 and 35.27 (average) for LCa and LCa + PME, respectively (*p* < 0.05). An increase in *b* ^*^ values was observed in all treatments (Figure 3F).

It was possible to observe an increase in pH values (*p* < 0.05) over time from the third day of storage of FC strawberries, with values of 3.77 for the first day and 3.91 and 3.96 for Days 3 and 6, respectively (Table 1). No differences in TA were observed between treatments, with average values of 0.97 g/100 g of citric acid (data not shown). An increase in SSC was observed on Day 3 only for the C treatment (Table 1). The treatments did not differ on the same day of analysis. The SSC/TA ratio is the relationship between the °Brix and the acidity of the product, being an indicator of quality (Couto and Canniatti‐Brazaca 2010). In this study, no significant differences were observed when evaluating the SSC/TA ratio with average values of 5.36 for all FC strawberries.

**TABLE 1:** Physico‐chemical analyses of fresh‐cut strawberry halves stored at 5°C ± 1°C.

	Treatment	Days		
		0	3	6
pH	C	3.8 ± 0.02 Ab	3.91 ± 0.01 Aa	3.96 ± 0.04 Aa
	WI	3.81 ± 0.04 Ab	3.95 ± 0.01 Aa	3.91 ± 0.02 Aa
	LCa	3.82 ± 0.03 Ab	3.92 ± 0.04 Aa	3.99 ± 0.01 Aa
	PME	3.73 ± 0.03 Ab	3.9 ± 0.01 Aa	3.88 ± 0 Aa
	LCa + PME	3.79 ± 0 Ab	3.93 ± 0.02 Aa	3.97 ± 0.01 Aa
SSC (°Brix)	C	4.73 ± 0.22 Ab	6.07 ± 0.05 Aa	5.33 ± 0.05 Aab
	WI	4.93 ± 0.6 Aa	5.07 ± 0.03 Aa	4.67 ± 0.12 Aa
	LCa	5.2 ± 0.52 Aa	5.3 ± 0.08 Aa	5.27 ± 0.11 Aa
	PME	4.93 ± 0.6 Aa	5.77 ± 0.27 Aa	5.23 ± 0.11 Aa
	LCa + PME	4.73 ± 0.22 Aa	5.27 ± 0.07 Aa	5.4 ± 0.08 Aa
SSC/TA	C	5.06 ± 0.22 Aa	6.04 ± 0.07 Aa	5.31 ± 0.12 Aa
	WI	5.53 ± 0.57 Aa	5.4 ± 0.21 Aa	4.95 ± 0.1 Aa
	LCa	5.64 ± 0.55 Aa	5.21 ± 0.14 Aa	5.17 ± 0.07 Aa
	PME	5.14 ± 0.72 Aa	5.77 ± 0.4 Aa	5.28 ± 0.19 Aa
	LCa + PME	4.99 ± 0.28 Aa	5.33 ± 0.27 Aa	5.53 ± 0.3 Aa
Anthocyanin (mg/100 g fresh fruit)	C	14.61 ± 0.44 Aa	5.09 ± 0.17 BCc	9.76 ± 0.31 Ab
	WI	13.36 ± 0.04 Aa	6.92 ± 0.35 Ac	8.23 ± 0.25 Bb
	LCa	13.96 ± 0.32 Aa	3.8 ± 0.29 Cc	5.71 ± 0.21 Cb
	PME	13.41 ± 0.19 Aa	3.85 ± 0.36 Cc	5.94 ± 0.46 Cb
	LCa + PME	13.66 ± 0.2 Aa	6.42 ± 0.37 ABb	4.83 ± 0.16 Cc

*Note*: C—no treatment and infusion; water—infusion with water; LCa—infusion with calcium lactate; PME—infusion with pectinmethylesterase; LCa + PME—infusion with calcium lactate and pectin methylesterase. Different uppercase letters indicate a statistical difference between treatments on the same day and different lowercase letters indicate a statistical difference between the same treatment across days using the Tukey test (*p* < 0.05).

Abbreviations: PME, pectin methylesterase; SSC, soluble solids content.

A reduction in anthocyanin content in the FC strawberries was observed over time (*p* < 0.05) (Table 1). Strawberries treated with infusion with water alone and LCa + PME showed higher anthocyanin levels when compared to the other treatments.

## 4 Discussion

### 4.1 Firmness

Firmness is one of the most important parameters that determine the acceptability and shelf life of fruits and vegetables (Goulao and Oliveira 2008). Calcium‐treated strawberries exhibited greater firmness over time. This is attributed to the association of calcium ions, which form cross‐links between pectin chains by binding to free carboxyl groups, thereby strengthening the cell wall (Udomkun et al. 2014). Additionally, the rapid formation of calcium‐pectate cross‐links during processing likely contributed to enhanced tissue cohesion and reduced softening immediately after cutting, thus explaining the significant differences in parameters such as firmness observed already at Day 0 among treatments.

The loss of firmness of strawberries treated only with PME may be associated with the absence of sufficient endogenous calcium ions to crosslink pectic polymers by binding to the free carboxyl groups created by PME, making the demethylated pectin polymers susceptible to degradation by other pectolytic enzymes, such as PG (Figure 5) (Chávez‐Sánchez et al. 2013; Lara et al. 2004). Therefore, the vacuum infusion of calcium (LCa and LCa + PME) was effective in maintaining the firmness of FC strawberries. These results corroborate those obtained by Yang et al. (2017), who found that the application of PME and calcium lactate increased the hardness of cut papaya. Tappi et al. (2016) also found that the infusion of calcium lactate in FC melon maintained the texture of the fruit. Vacuum impregnation with calcium in Kyoho grapes resulted in an increase in firmness from 12.93 to 14.4 N during storage (Mao et al. 2017). Thus, the availability of calcium ion through infusion with calcium lactate maintains the structure since the methyl groups of hydrolyzed pectin bind adjacent pectin polymers, making the cell wall firmer (Kirtil et al. 2014). Therefore, the application of LCa and LCa + PME can be an efficient alternative in improving the firmness of FC strawberries.

### 4.2 Weight Loss

Water loss is one of the factors that can affect the texture of fruits and vegetables as it leads to loss of turgor and crunchiness, giving the product a withered appearance and flabby texture, which can harm its market value and acceptability by the consumer (Prajapati et al. 2021). Furthermore, water loss tends to occur more quickly in FC products due to the absence of epidermal layers and the exposure of internal tissues caused by tissue rupture during the cutting and peeling operations, which was observed in all treatments from Day 3 (Rolle and Chism 1987; Toivonen and Brummell 2008). However, excessive weight loss in PME treatments may be associated with physiological processes, such as respiration, throughout the storage period, and enzymatic activity, as an increase in respiratory rate, and higher values of enzymatic activity were observed in relation to calcium treatments (Figure 2) (Ndukwu and Chinenye 2011). These results corroborate those found by Zhang et al. (2019), who found a positive effect on the weight loss of jujube fruit treated with vacuum infiltration of Ca + PME and vacuum infiltration of Ca. Therefore, the application of the vacuum infusion of calcium lactate combined with refrigerated storage of FC strawberries in the current work contributed to maintaining quality and reducing weight loss.

### 4.3 Electrolyte Efflux

With the increase in enzymatic activity and substantial changes in firmness, changes occur in the cellular structure of FC fruit. Thus, an increase in electrolyte leakage was observed on the third day of storage for FC strawberries (Figure 3). This was likely due to a loss of membrane integrity that allowed effusion of cellular osmotic solutes into the apoplastic space, which resulted in water movements out of the tissue and loss of turgor (Toivonen and Brummell 2008).

The action of PME enables the formation of a complex of calcium and pectin, providing increased cell wall rigidity and tissue firmness, which was verified in this study in treatments with calcium ion, especially the treatment with LCa + PME, which showed less electrolyte efflux in relation to the control on the Day 6 (Aghdam et al. 2012). This study is in line with that of Ngamchuachit et al. (2014) who found a less effusion of electrolytes in FC mangoes treated with a calcium source, promoting a stabilizing effect. Similarly, Phanumong et al. (2016) found that calcium immersion treatment reduced electrolyte efflux when compared to the control in FC lychees. In the present study, a lower percentage of electrolyte leakage from FC strawberries subjected to infusion with LCa + PME was evidenced on the last day of storage, indicating that the use of these substances can help maintain the structural integrity of the FC strawberry tissues.

### 4.4 Respiratory Rate

The results indicate that treatments with a source of calcium promoted the lowest respiratory rate among the treatments applied. This effect can be attributed to the role of calcium in the physiological pattern of fruits, where the reduction in cell wall degradation resulting from the formation of the calcium–pectin bonds and pectin crosslinking results in a less solubilization of substrates necessary for enzyme activities, including those involved in the respiratory process, consequently decreasing the respiratory rate (Batista‐Silva et al. 2018; Kirtil et al. 2014). This enhanced tissue cohesion, evident from Day 0, can also influence gas diffusion and result in a slightly different respiration pattern. The results corroborate those found by Perez‐Cabrera et al. (2011) when evaluating the effect of infusing FC pears with salts, they found that impregnation with calcium lactate had the most notable effect in that the rate of oxygen consumption was reduced compared to untreated samples, as was the rate of CO_2_ generation. Luna‐Guzmán et al. (1999) also found that calcium decreased metabolism as it reduced the respiratory rate of FC melon; in addition, calcium salts maintained the firmness of the melons throughout refrigerated storage.

However, an increase in the respiratory rate was observed on Day 6. This increase may be associated with microbial activity as the presence of rot (3%) was verified in the samples on the last day of storage. Luna‐Guzmán et al. (1999) observed that cutting and slicing makes FC products more prone to microbial contamination. FC processing also increases enzymatic degradation (Rybak et al. 2020). This is due to the increased exposed surface area that allows oxygen to diffuse more quickly into the cells and the increased metabolic activity of injured cells (Zagory 1995). Therefore, infusion with LCa and PME can be an effective approach for reducing the respiratory rate and consequently maintaining the quality of FC strawberries.

### 4.5 DM and Galacturonic Acid

The DM provides an estimate of the carboxyl groups that are methylated, thus not available to bind with calcium ions (Ciriminna et al. 2022). Thus, an increase in the DM can reduce the formation of calcium bridges and consequently destabilize pectins (Lara et al. 2004). In this study, a higher DM was verified for treatments with LCa + PME, indicating less availability for the formation of the pectin network, with a reduction in the values of the DM in the subsequent days. Consequently, firmness increased on these days, indicating that calcium ions may have bonded to unmethylated galacturonic acid residues forming the calcium bridge (Lara et al. 2004). Langer et al. (2019), when evaluating the effects of calcium on the metabolism of the strawberry cell wall, observed that the calcium treatment demonstrated a lower value in the degree of esterification of pectins when compared to the control and was verified for the LCa treatment +PME in the first days of storage. Therefore, a connection with calcium may have occurred, helping to improve the firmness of minimally processed strawberries.

### 4.6 PME Activity

The loss or increase in fruit firmness is directly linked to the activity of the enzymes responsible for pectin degradation (Posé et al. 2019). During storage, PME activity increased in strawberries, due to the rupture of the cell wall during cutting, which releases pectolytic enzymes that promote the breakdown of pectins and compromise the structure of the fruits (Toivonen and Brummell 2008; Yang et al. 2017). It was shown that vacuum infusion with PME may have accelerated the impregnation of enzymes in the fruits, resulting in greater PME activity (Guillemin et al. 2006). Furthermore, the increase in enzyme activity in water treatment may indicate a negative effect of PME Infusion in strawberries, whereas in calcium treatments this effect was not observed. With the increase in enzymatic activity, there was a loss of firmness for treatments with and without infusion without the application of LCa. Anjongsinsiri et al. (2006) and Guillemin et al. (2006) who verified an increase in PME activity after vacuum infusion of strawberries and apples respectively.

However, an improvement in the firmness of the LCa and PME + LCa samples was verified, which may be associated with the response of the PME action with the availability of calcium in solution, which allows the calcium ion to bind to the pectin molecules that make up the cell wall, thus extending fruit firmness (Huang et al. 2023). This immediate and efficient cross‐linking during initial processing may significantly contribute to the enhanced tissue cohesion observed from Day 0, possibly explaining the early differences in firmness among treatments. Similar results were found by Sirijariyawat et al. (2012), when applying PME and calcium by infusion, they found that the PME activity of mango cubes was significantly higher after infusion at all pressure levels. Langer et al. (2019) observed that calcium treatment increased the activity of PME while inhibiting those corresponding to pectin hydrolases such as PG and β‐galactosidase. Therefore, vacuum infusion with LCa + PME can be effective in maintaining or improving the quality of FC strawberries, promoting the formation of calcium pectate and inhibiting the activity of pectolytic enzymes.

### 4.7 Visual Quality (Visible Spoilage, Freshness, and Damage)

A reduction in freshness and an increase in the percentage of damage were observed mainly in the groups treated with PME. Weight loss is a crucial factor in the quality and commercialization of vegetable products, as it directly affects firmness and appearance, which can lead to wilting of the product (El‐Beltagi et al. 2022). In this study, an increase in water loss was observed over time in all treatments, being more significant in the group treated with PME. This treatment also presented the highest percentage of damage and loss of freshness throughout the storage period, differing from the samples treated with LCa + PME on the sixth day.

The presence of calcium can inhibit the activity of endogenous PME, preserving the structure of the strawberry cell wall. The endogenous calcium present in PME treatments may have been insufficient to promote this inhibition, resulting in reduced fruit firmness (Kirtil et al. 2014). Vacuum infusion of enzymes and calcium leads to the formation of calcium pectate, which improves and maintains fruit firmness, preventing damage (Carnelossi et al. 2018).

The low incidence of rot observed (3%) can be attributed to the possible microbial resistance promoted by LCa, as it is an organic acid salt, and to mechanical resistance, as mechanical damage can be a means of entry for microorganisms (Aguayo et al. 2015; Khademi and Khoveyteri‐Zadeh 2022). For example, Phanumong et al. (2016) evaluated the application of various calcium salts on the quality and texture of FC lychee fruits and verified an increase in the product's shelf life to 9 days on the basis of sensory scores. It was found in minimally processed jackfruit that the application of calcium lactate with vacuum infusion showed positive results in reducing contamination during storage compared to control and that vacuum infusion prolonged shelf life (Sandianysamy et al. 2023). Nasri et al. (2023), when evaluating the effect of calcium lactate and ultrasound on mushrooms, observed a significant reduction in contamination. Therefore, infusion with LCa and PME appears as an effective alternative in maintaining freshness and reducing damage in FC strawberries during storage.

### 4.8 Color

The tendency toward a reduction in luminosity parameters observed in treatments over time may be associated with an increase in vacuum, which in turn leads to a greater impregnation of the solution and greater gas–liquid exchange, promoting changes in color coordinates, which may cause changes in the refractive index (Tapia et al. 1999). The index *a* ^*^ represents the color from green to red, which may reflect the degree of maturation; however, no variation was observed over time. Likewise, Tappi et al. (2016), when evaluating minimally processed melon with calcium lactate vacuum impregnation, observed a reduction in *L* ^*^ (luminosity) in all treatments applied. Yang et al. (2017) when evaluating the vacuum impregnation of calcium lactate and PME in minimally processed papaya, they found a decrease in the *L* ^*^ and *C* ^*^ index in all treatments as a result of pigment degradation; in addition, they found that the infusion was able to delay the degradation of pigments once the gaseous phase of the fruit was removed. Perez‐Cabrera et al. (2011) found that calcium lactate applied to freshly vacuum‐processed pears maintained their color and firmness during storage. Thus, the joint and efficient action of PME and calcium allows the structure of the cell wall to remain unchanged, maintaining the color and visual characteristics of FC strawberries. These effects are directly related to fruit freshness perception by consumers, as color stability is a key indicator of ripeness and overall visual quality. This relationship is supported by the results in Figure 3A, which showed reduced freshness and increased damage mainly in PME‐treated groups, whereas LCa + PME preserved visual quality, aligning with the maintenance of color and structural integrity described above.

### 4.9 pH

The observed pH increase across treatments from Day 3 of storage likely reflects cellular damage responses during minimal processing. Cutting induces pH changes in FC fruit: immediate acidification from vacuolar content leakage (lowering pH), followed by progressive alkalinization due to metabolic shifts, including ammonia release from deamination reactions and membrane lipid degradation (Beaulieu and Gorny 2002; Toivonen and Brummel 2008). Furthermore, lower values observed in PME treatments may indicate the loss of cellular content. Results that corroborate this finding were reported by Sandianysamy et al. (2023) who, when evaluating the infusion of LCa in minimally processed jackfruit, verified the same behavior, where an increase in pH was observed after the application of treatment and during storage.

### 4.10 SSC, Titratable Acidity (TA), and SSC/TA Ratio

After harvest and during storage, there is a tendency for fruits to decrease in the quantities of the main precursors of respiratory metabolism, such as sugars and acids. However, in this study, as the respiratory rate was stable until Day 3 of storage, no significant variations in these parameters were observed, in agreement with Zhang et al. (2008). The SSC is an important quality index related to fruit flavor that develops during ripening, namely, sweetness. Despite the increase in SSC being observed from Day 3 onwards, the treatments applied did not differ from each other. Thus, this may indicate that the treatments do not cause significant changes in this quality parameter, as the greater the decline in SSC, the greater the degradation in fruit quality (Li et al. 2015). Zhang et al. (2019), when evaluating the effects of methylesterase and calcium on the quality attributes of vacuum‐impregnated jujube fruit, found that the application of Ca and Ca + PME by vacuum infiltration could preserve the quality of the jujubes during storage. On the other hand, Sun et al. (2023), when evaluating changes in the forms of Ca and soluble pectin in chelate of cherry tomatoes treated with ultrasound‐assisted calcium lactate, found no differences in the levels of SSC between treatments. Furthermore, when evaluating the SSC/TA ratios, no differences were found between treatments. The SSC/TA can be an indication of the degree of ripeness of many fruits, being a parameter used to indicate a possible pleasant sensation on the consumer's palate; however, this parameter proved to be stable during storage of guava fruit (Azzolini et al. 2004). Thus, we can conclude that the organic acid content and SSC of the FC strawberries and the SSC/TA do not undergo significant changes, causing no noticeable sensory changes, as the products remained stable. Thus, the results suggest that the application of the LCa and LCa + PME treatments in the current work maintain taste the characteristics of the product.

### 4.11 Anthocyanin

The anthocyanin content of strawberries is a key quality attribute, affecting both visual appeal (red color intensity) and antioxidant capacity, but is highly vulnerable to postharvest handling (Crecente‐Campo et al. 2012; Nunes et al. 2006). In our study, anthocyanin levels declined by Day 3 in all treatments, consistent with earlier reports that FC processing accelerates pigment degradation due to increased surface area enhancing oxygen diffusion and promoting anthocyanin oxidation (Zagory 1995), cutting injury upregulates polyphenol oxidase (PPO) and peroxidase (POD), as observed by Van de Velde et al. (2018) in FC strawberries and cellular damage altering apoplastic pH, which destabilizes anthocyanins (Gil et al. 1997; Odriozola‐Serrano et al. 2009). However, the subsequent anthocyanin rebound by Day 6 (Table 1) suggests that though weight loss was statistically insignificant (Figure 1B), even marginal moisture evaporation could concentrate cellular pigments, artificially elevating spectrophotometric readings. The stress may trigger anthocyanin synthesis via the upregulation of phenylalanine ammonia‐lyase (PAL) (Van de Velde et al. 2018). Additionally, the observed increase in pH could facilitate conversion of colorless chalcones to colored anthocyanidins and shift equilibrium toward more stable flavylium cations at moderately acidic pH, although this effect may have been mitigated by calcium‐induced pigment stabilization in the LCa treatment (Khoo et al. 2017). Therefore, studies are needed to further evaluate the effect of the treatments evaluated here on FC strawberries.

## 5 Conclusion

The infusion of calcium lactate into FC strawberry halves proved highly effective in preserving fruit quality and extending shelf life by up to 3 days. Vacuum infiltration with calcium lactate increased firmness by 119.8% on Day 3, whereas the combination with PME further enhanced firmness by 90.7%, reduced electrolyte efflux by 38% compared to the control, and maintained respiration rates significantly lower than untreated samples. Both treatments delayed weight loss to below 0.8% (except PME alone), preserved freshness and anthocyanin content, and minimized fruit damage, highlighting their potential for improving post‐harvest quality in the FC fruit industry.

## Author Contributions

**Alysson Caetano Soares**: conceptualization, investigation, writing – original draft, methodology, validation, writing – review and editing, formal analysis, data curation, software. **Mônica Silva de Jesus**: formal analysis, methodology. **Steven A. Sargent**: writing – review and editing, writing – original draft, visualization. **Jeffrey K. Brecht**: writing – original draft, writing – review and editing, visualization. **Luiz Fernando Ganassali de Oliveira Junior**: writing – original draft, writing – review and editing, validation. **Marcelo Augusto Gutierrez Carnelossi**: writing – original draft, writing – review and editing, visualization, methodology, supervision, conceptualization, project administration, data curation.

## Conflicts of Interest

The authors declare no conflicts of interest.
